# Nardoguaianone L Isolated from *Nardostachys jatamansi* Improved the Effect of Gemcitabine Chemotherapy via Regulating AGE Signaling Pathway in SW1990 Cells

**DOI:** 10.3390/molecules27206849

**Published:** 2022-10-13

**Authors:** Yi-Dan Zheng, Li-Mei Ma, Jin-Jian Lu, Tian Chai, Mohammad Reza Naghavi, Jun-Yi Ma, Chun-Yan Sang, Jun-Li Yang

**Affiliations:** 1 CAS Key Laboratory of Chemistry of Northwestern Plant Resources and Key Laboratory for Natural Medicine of Gansu Province, Lanzhou Institute of Chemical Physics, Chinese Academy of Sciences, Lanzhou 730000, China; 2 College of Life Science, Northwest Normal University, Lanzhou 730070, China; 3 University of Chinese Academy of Sciences, Chinese Academy of Sciences, Beijing 100049, China; 4 State Key Laboratory of Quality Research in Chinese Medicine, Institute of Chinese Medical Sciences, University of Macau, Macao, China; 5 Agricultural & Natural Resources College, University of Tehran, Karaj 3158777871, Iran

**Keywords:** pancreatic cancer, gemcitabine, proteomic, AGE-RAGE signaling pathway, SW1990 cells

## Abstract

Pancreatic cancer is the seventh leading cause of cancer-related death worldwide and is known as “the king of cancers”. Currently, gemcitabine (GEM) as the clinical drug of choice for chemotherapy of advanced pancreatic cancer has poor drug sensitivity and ineffective chemotherapy. Nardoguaianone L (G-6) is a novel guaiane-type sesquiterpenoid isolated from *Nardostachys jatamansi* DC., and it exhibits anti-tumor activity. Based on the newly discovered G-6 with anti-pancreatic cancer activity in our laboratory, this paper aimed to evaluate the potential value of the combination of G-6 and GEM in SW1990 cells, including cell viability, cell apoptosis, colony assay and tandem mass tags (TMT) marker-based proteomic technology. These results showed that G-6 combined with GEM significantly inhibited cell viability, and the effect was more obvious than that with single drug. In addition, the use of TMT marker-based proteomic technology demonstrated that the AGE-RAGE signaling pathway was activated after medication-combination. Furthermore, reactive oxygen species (ROS) and mitochondrial membrane potential (MMP) assays were used to validate the proteomic results. Finally, apoptosis was detected by flow cytometry. In conclusion, G-6 combined with GEM induced an increase in ROS level and a decrease in MMP in SW1990 cells through the AGE-RAGE signaling pathway, ultimately leading to apoptosis. G-6 improved the effect of GEM chemotherapy and may be used as a potential combination therapy for pancreatic cancer.

## 1. Introduction

Pancreatic cancer is a malignant tumor of digestive tract with poor prognosis and increasing incidence year by year, and its 5-year survival rate is less than 5%; the median survival time after diagnosis is about 6 months [1]. Previous experience showed that surgical resection is the best way to cure pancreatic cancer. Unfortunately, pancreatic cancer is occult in the early stage and not easy to be diagnosed. Moreover, 80–85% of patients were diagnosed at an advanced stage and not eligible for resection [2]. In addition, more than 90% of patients may die of recurrence after surgery without additional treatment [3]. According to the above characteristics of pancreatic cancer treatment, chemotherapy is still the main treatment for pancreatic cancer. Gemcitabine (GEM) is currently the first-line drug for clinical chemotherapy in pancreatic cancer. However, much more effective therapy is highly desirable [4,5]. Currently, combined chemotherapy is the main treatment for advanced pancreatic cancer [6,7]. The synergistic effects of GEM are improved by natural products; for example, the co-treatment with gemcitabine and herbal mixture extract C5E was more effective than each individual treatment [8]. Multiple natural products combined with GEM achieved more potent anti-pancreatic cancer properties through enhanced bioavailability or reduced toxicity [9]. Therefore, finding a combination therapy for pancreatic cancer is an urgent problem to be solved. Natural products have displayed great potential in pancreatic cancer therapy [10,11,12,13,14]. *Nardostachys jatamansi* DC. is an endangered perennial herbal medicinal plant, and has anti-tumor, anti-depressant, anti-oxidant and other pharmacological activities [15,16]. G-6 is a novel guaiane-type sesquiterpenoid extracted from the roots and rhizomes of *Nardostachys jatamansi* by our research group, and the related spectroscopic data were exhibited in supplemental materials Figures S1 and S2 [17].

Advanced glycation end-products (AGEs) are a group of stable end products, which produced from a variety of different intermediates. The receptor pathway is the main pathway of AGEs, and the most studied receptor is the receptor for advanced glycation endproducts (RAGE) [18]. RAGE is expressed in cancer cells, such as pancreatic cancer and prostate cancer, and is closely related to oxidative stress in the body [19,20]. The development of drug-resistant pancreatic cancer can be accelerated by RAGE-initiated signaling. Reactive oxygen species (ROS) increase when the body is stimulated by oxidative stress, and the accumulation of ROS will destroy the ability of the anti-oxidant system. As bi-functional molecules, cancer cells are more vulnerable to ROS than normal cells [21]. In addition, ROS can also kill cancer cells by inducing apoptosis [22,23]. Therefore, AGE-RAGE signaling pathway is considered to be closely related to cancer therapy. In conclusion, G-6 is expected to be an effective candidate combined with GEM to inhibit the growth of pancreatic cancer.

## 2. Results

### 2.1. Toxicity of G-6 Combined with GEM on Four Pancreatic Cancer Cell Lines

**Figure 1:**
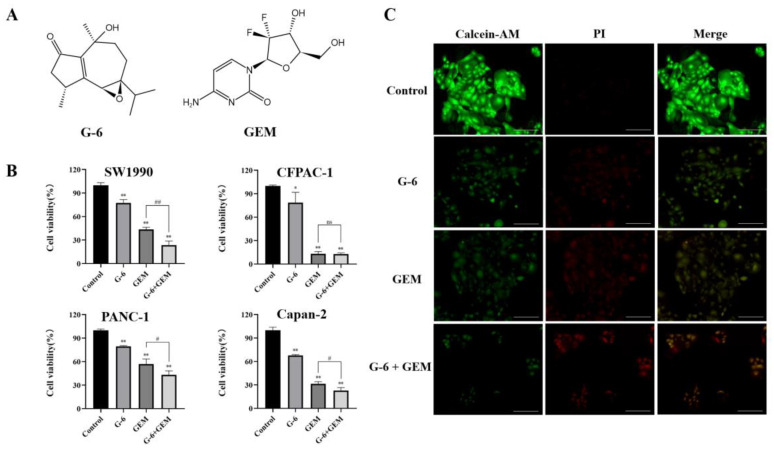
Effect of G-6 and GEM on proliferation of pancreatic cells. (**A**) Chemical structure of G-6 and GEM. (**B**) G-6 (40 μM), GEM (5 μM), respectively or combined in SW1990, CFPAC-1, Capan-2, and PANC-1 cells for 72 h, and the cell viability was detected by MTT. (**C**) Living and dead cells were observed with Calcein/PI co-staining. Data are presented as mean ± SD (*n* = 3). * *p* < 0.05, ** *p* < 0.01 compared with the control; # *p* < 0.05, ## *p* < 0.01, ns indicates not statistically significant, G-6 + GEM compared with GEM. Scale bar = 100 µm.

The chemical structures of G-6 and GEM are shown in Figure 1A. In order to study the toxicity of G-6 combined with GEM against pancreatic cancer cells, the MTT assay was carried out. SW1990, CFPAC-1, Capan-2, and PANC-1 cells are all derived from the human body and have an epithelioid morphology with a high degree of malignancy. The results showed that G-6 combined with GEM inhibited the survival of SW1990 cells by 1.35- to 3.39-fold but had no obvious inhibitory effect on CFPAC-1 cells and inhibited the survival rate of Capan-2 cells by 1.12- to 2.39-fold and that the survival rate was inhibited by 1.32- to 2.83-fold in PANC-1 cells (Figure 1B). Therefore, it was concluded that the combination drug was most susceptibility in SW1990 cells, which were selected for subsequent experiments. Further, the effect of G-6 combined with GEM on the survival and death of SW1990 cells was detected with calcein/PI co-staining. It can be seen from Figure 1C that the green color represented the live cells stained by calcein, and the staining degree gradually weakened. The red represented dead cells stained with PI, and the color gradually deepens, which indicated that G-6 combined with GEM had certain toxicity to SW1990 cells.

### 2.2. G-6 Combined with GEM Inhibited the Growth of SW1990 Cells

**Figure 2:**
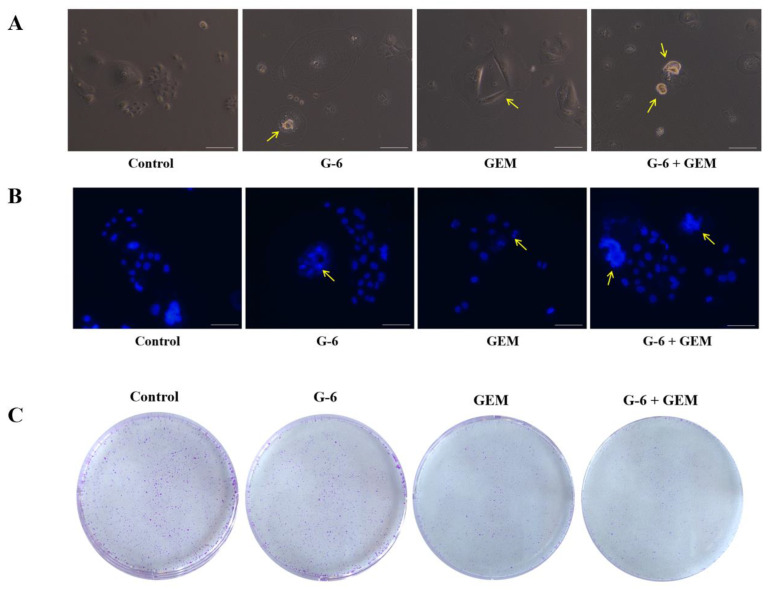
Inhibitory effect of G-6 combined with GEM in SW1990 cells. (**A**) Morphological observation of G-6 combined with GEM in SW1990 cells. Arrows indicate abnormal cells. (**B**) The nucleus of SW1990 cells were observed by Hoechst 33342 staining. Arrows indicate abnormal nucleus. (**C**) Image of colony formation in SW1990 cells. Scale bar = 100 µm and 200 µm.

The microscopic observation of the cell morphology and colony formation indicated that G-6 combined with GEM inhibited the growth of SW1990 cells. Cells of control group had a clear three-dimensional shape and a round shape, and cell nuclear chromatin was evenly distributed. The morphology of cells changed after G-6 and GEM treatment. After combined treatment, the cells became brighter and dirtier, and the cell exhibited nuclear agglutination and nucleolar contraction (Figure 2A,B). Colony formation assays can reflect the proliferative capacity and population dependence of cells. Compared with the control group or single drug group, colony formation numbers were greatly reduced after G-6 combined with GEM treatment (Figure 2C).

### 2.3. Proteomic Comparison of G-6 Combined with GEM in SW1990 Cells

**Figure 3:**
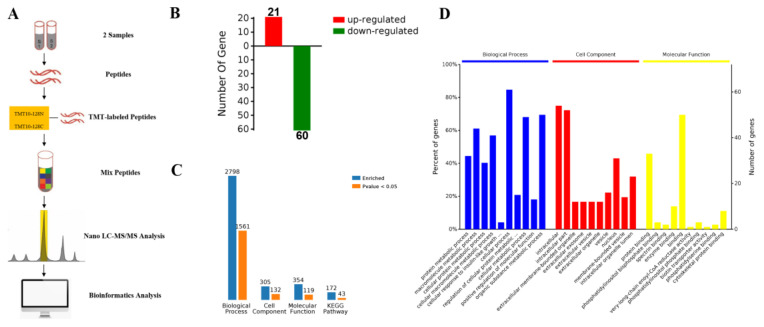
TMT-labeled proteomics analysis. (**A**) The general flow of proteomic analysis. Proteins in SW1990 cells treated with 128N and 128C TMT were labeled with GEM and combined G-6 and GEM, respectively. Labeled proteins were analyzed by LC-MS/MS and differentially expressed proteins were analyzed by bioinformatics. (**B**) Differentially expressed proteins were obtained by sample analysis. (**C**) The differentially expressed proteins were enriched and analyzed. (**D**) GO enrichment analysis.

SW1990 cells were treated with GEM (5 µm) and G-6 (40 µm) for 48 h and proteins were extracted, labeled with TMT tags, and analyzed by LC-MS/MS (Figure 3A). A total of 81 differentially expressed proteins were identified, including 21 up-regulated proteins and 60 down-regulated proteins (Figure 3B). Further, differentially expressed proteins were analyzed by means of bioinformatics. From GO analysis, protein enrichment for biological process (BP), cellular component (CC), and molecular function (MF) was obtained (Figure 3C). Among the BPs, there were a total of 2798 BPs, of which 1561 were significantly different. It mainly included protein metabolic process and macromolecule metabolic process. Among the CCs, there were a total of 305 BPs, of which 132 were significantly different. CC analysis showed that most of the identified proteins were intracellular. Among the MFs, there were a total of 354 BPs, of which 119 were significantly different. Molecular function results revealed that most of these proteins had binding activity (Figure 3C,D).

### 2.4. KEGG Pathway and Protein-Protein Interaction Network (PPI) Analysis

**Figure 4:**
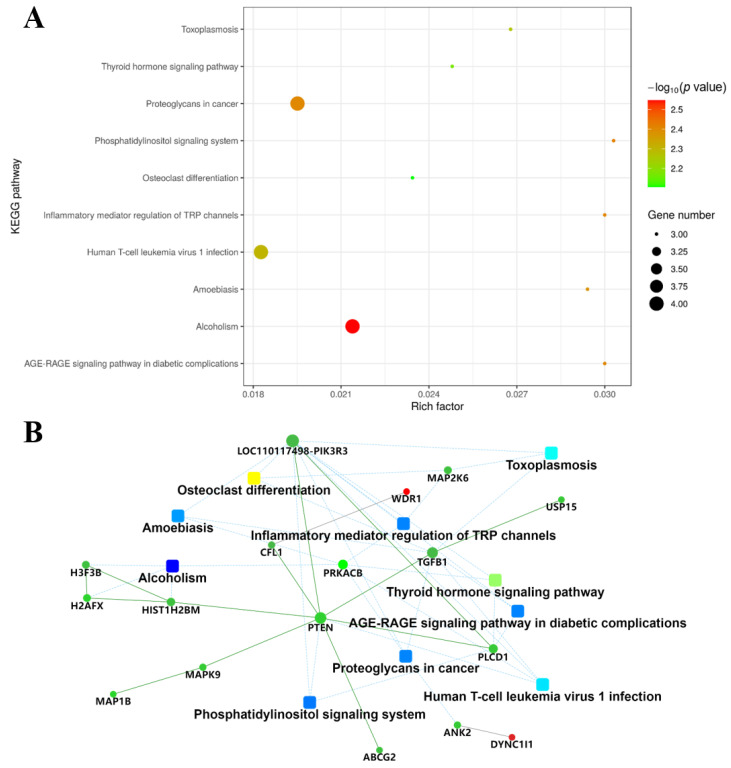
KEGG pathway and PPI analysis. (**A**) Pathway bubble chart of the top 10 results of differential protein KEGG enrichment. (**B**) PPI analysis was performed based on factors such as fold change of differentially expressed proteins, enrichment of the KEGG pathway, and biological processes.

A total of 172 KEGG pathways were enriched, of which 43 were significantly different, and the 10 with the greatest differences are listed in Figure 4A. These pathways included the AGE-RAGE signaling pathway (hsa04933), thyroid hormone signaling pathway (hsa04919), inflammatory mediator regulation of TRP channels (hsa04750), alcoholism (hsa05034), phosphatidylinositol signaling system (hsa04070), proteoglycans in cancer (hsa05205), amoebiasis (hsa05146), human T-cell leukemia virus 1 infection (hsa05166), toxoplasmosis (hsa05145), and osteoclast differentiation (hsa04380). PPI analysis could further dissect and exhibit the acquired protein data. In the network, square referred to GO/KEGG term, circle referred to Protein/Gene. Red circles indicated that the protein expression was up-regulated, and the green circles was down-regulated. Proteins were down-regulated in various functional pathways. PI3K, TGF*β*1 and PLCD1 were all down-regulated in the ARE-RAGE signaling pathway (Figure 4B).

### 2.5. Evaluation of TMT Results Using ROS Assay and MMP Assay

**Figure 5:**
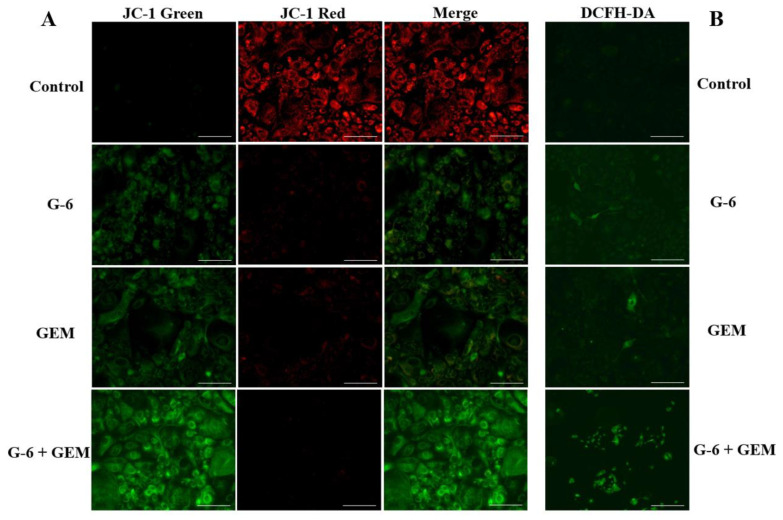
Representative fluorescent images of intracellular MMP and ROS assays. (**A**) Combined use of G-6 and GEM disrupted MMP in SW1990 cells. (**B**) Combined use of G-6 and GEM increased ROS in SW1990 cells. Scale bar = 100 µm.

Based on the above proteomic analysis, ARE-RAGE signaling pathway might be associated with the antitumor activity of SW1990 after the combined use of G-6 and GEM. This pathway is regulated to increase ROS [18,19]. However, the accumulation of ROS is thought to be related to the disruption of MMP [24]. MMP and ROS changes of cells were expressed by JC-1 and DCFH-DA fluorescent probe, respectively. As shown in Figure 5A, the fluorescence images showed that the control group cells were predominantly red. In contrast, cells treated with G-6 and GEM combined had the strongest green fluorescence and the weakest red fluorescence, indicating that MMP was decreased. On the other hand, the fluorescence images showed that the combined G-6 and GEM-treated cells exhibited the highest fluorescence intensity compared with the control group, indicating the accumulation of ROS (Figure 5B).

### 2.6. G-6 Combined with GEM Induced Apoptosis in SW1990 Cells

**Figure 6:**
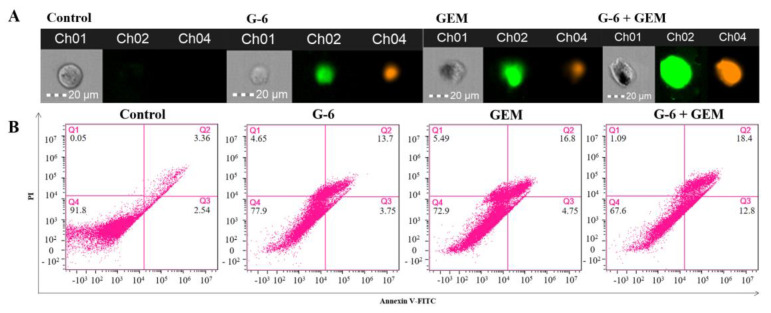
G-6 combined with GEM induced apoptosis in SW1990 cells. (**A**) Representative fluorescent images of Annexin V/PI co-staining in SW1990 cells. (**B**) Annexin V/PI co-staining was used to detect apoptosis rate. Scale bar = 20 µm.

To determine whether the combination of G-6 and GEM induced apoptosis of SW1990 cells, the apoptosis rate was detected by Annexin V/PI co-staining. In the control group, cells could not be stained by FITC and PI. As shown in Figure 6A, cells treated with G-6 and GEM, respectively, showed green and orange-red. The staining degree was deepened after combined treatment. These are typical early and late apoptosis morphological features. Subsequently, the apoptosis rate induced by G-6 and GEM was detected by flow cytometry. Apoptosis rates gradually deepened to 17.45%, 21.55%, and 31.2% (Figure 6B). These results indicated that G-6 and GEM could induced apoptosis in SW1990 cells.

## 3. Discussion

Most naturally occurring compounds have various bioactivities and few side effects [11]. In the past 30 years, 80% of FDA-approved cancer treatments are natural products or their derivatives [25]. Among them, sesquiterpenoids, as a group of low-toxicity small molecules, have various biological activities such as anti-tumor and anti-inflammatory [26,27]. Previous research has shown that guaiane-type sesquiterpenoids from *Artemisia atrovirens* had cytotoxicity against HepG2, SMMC-7721, and Huh7 cells [28]. Increasing evidence indicates that combination therapy is a promising strategy to enhance treatment outcomes and improve patient outcomes [29,30,31,32]. Therefore, the combination of phytochemicals with conventional chemotherapeutic drugs attracted extensive attention in recent years. As a guaiane-type sesquiterpenoid, G-6 is isolated from *Nardostachys jatamansi* DC. and determined by our research group for the first time, as well as shows anti-tumour potential for development [17].

TMT-labeled proteomics technology is a new isotope labeling technology combined with tandem mass spectrometry [33,34]. According to the bioinformatics analysis, proteins that influence cancer cell viability can be identified in cancer therapy. Herein, we used quantitative TMT-labeled proteomic techniques to identify proteome-level changes in SW1990 cells treated by G-6 combined with GEM compared to GEM only. There were 81 differentially expressed proteins identified, of which 21 were up-regulated and 60 were down-regulated (Tables S1 and S2). Bioinformatics analysis demonstrated that the functions of these differential proteins mainly involved protein metabolic process and macromolecule metabolic process, and the main signaling pathway involved is AGE-RAGE signaling pathway, which is activated by down-regulation of PI3K, TGF*β*1 and PLCD1 (Figure S3). The above results provided the experimental basis for elucidating the anti-tumor mechanism of G-6 combined with GEM in the treatment of SW1990 cells.

The AGE-RAGE signaling pathway is considered to be closely related to the oxidative stress and the accumulation of ROS [18]. Compared with normal cells, cancer cells have higher levels of ROS and oxidative stress [35]. Interestingly, ROS can inhibit the proliferation of cancer cells and even induce apoptosis after breaking a certain threshold [36]. In addition, the accumulation of ROS can lead to the decrease of MMP. Furthermore, the destruction of MMP can aggravate oxidative stress [37]. The four pancreatic cancer cells selected in this experiment can be inhibited by G-6 and GEM to varying degrees. According to the inhibitory effect, the most susceptible SW1990 cells were selected for further research.

Herein, JC-1 and DCFH-DA staining were used to examine the changes in intracellular MMP and ROS. G-6 combined with GEM increased ROS and decreased MMP in SW1990 cells compared with GEM administration only, and that also validated the proteomics results. Additionally, the MTT assay, morphological observation, calcein/PI co-staining, and colony formation assay were carried out to detect the cell viability, cytotoxicity, cell proliferation ability, and population dependence, respectively. Finally, apoptosis was detected by flow cytometry. These results indicated that G-6 combined with GEM increased the anti-tumor activity against SW1990 cells compared with GEM administration only. 

## 4. Materials and Methods

### 4.1. Chemicals and Reagents

Nardoguaianone L (G-6) was received from the CAS Key Laboratory of Chemistry of Northwestern Plant Resources, and the HPLC purity level was >98%. Gemcitabine (GEM, purity = 99.33%) was purchased from MedChemExpress.

### 4.2. Cell Culture and Grouping

Four human pancreatic cancer lines (SW1990, CFPAC-1, Capan-2 and PANC-1) were procured from the cell bank of the Chinese Academy of the Chinese Academy of Sciences (Shanghai, China). SW1990, CFPAC-1, Capan-2 and PANC-1 cells were cultured in Dulbecco’s modified Eagle’s medium (DMEM, Gibco, Carlsbad, CA, USA) comprising 10% fetal bovine serum (FBS, Sijing, Hangzhou, China) at 37 °C in 5% CO_2_ incubator. 0.25% Trypsin-EDTA (Gibco, Carlsbad, CA, USA) was used by passaging, and logarithmic growth phase cells were used in all experiments. The experimental groups were control group (without any treatment), G-6 treatment group (40 μM), GEM treatment group (5 μM), G-6 and GEM combined group (40 μM + 5 μM).

### 4.3. Cell Viability Assay [38]

MTT (Solarbio, Beijing, China) assay was showed to detect cell viability. SW1990, CFPAC-1, Capan-2 and PANC-1 cells (3 × 10^3^/well) were seeded into 96-well plates. After overnight, each group treating for 72 h, old medium was thrown away and added 100 μL of medium containing 10 μL of MTT solution to each well and further cultured for 4 h. The optical density (OD) of the samples was measured at 570 nm using a Microplate Reader (Rayto, Shenzhen, China). The cell viability (%) = (OD_570_ of treated group − OD_570_ of blank group)/(OD_570_ of untreated group − OD_570_ of blank group) × 100%.

### 4.4. Cytotoxicity Assay

Cells were (3 × 10^4^/well) were plated onto 96-well plates. After overnight and treating for 48 h, live and dead cells were detected by Calcein/PI cell Viability Assay Kit (Beyotime, Shanghai, China) and then observed by Mshot inverted fluorescence microscope (Guangzhou, China). Among them, live cells are stained green by calcein and dead cells are stained red by PI.

### 4.5. Colony Formation Assay [39]

Cells (1 × 10^3^/well) were seeded into a 6-well plates. After 10 days of treatment, colonies were rinsed with PBS and then fixed with 4% paraformaldehyde (Solarbio, Beijing, China) for 20 min. Finally, stained with crystal violet (Solarbio, Beijing, China) for 30 min before observation.

### 4.6. MMP Detection

Cells were (1 × 10^5^/well) were coated at 6-well plates. After overnight and treating for 48 h, according to the kit (Solarbio, Beijing, China) instructions, JC-1 was added for 20 min at incubator and cells were washed two times. Finally, staining was examined using the Mshot inverted fluorescence microscope (Guangzhou, China).

### 4.7. Intracellular ROS Detection Analysis

Intracellular ROS levels were detected using a ROS assay kit (Beyotime, Shanghai, China). Cells (1 × 10^5^/well) were seeded into a 6-well plates. According to the manufacturer’s instructions, DCFH-DA (1:1000) was diluted with FBS-free DMEM. After 48 h, 1 mL of diluted DCFH-DA was added and incubated at 37 °C for 20 min. Then, the cells were washed with FBS-free DMEM and photographed. ROS fluorescence images were observed with Mshot inverted fluorescence microscope (Guangzhou, China).

### 4.8. Nucleus Staining

Hoechst 33342 was used as a nucleus dye. Cells (3 × 10^3^/well) were seeded into a 96-well plates, After treatment for 48 h, cells were rinsed with PBS and then fixed with 4% paraformaldehyde (Solarbio, Beijing, China) for 20 min. Cells were stimulated with 0.3% Triton X-100 (Solarbio, Beijing, China) for 20 min. Finally visualized after 3 min of Hoechst 33342 (Solarbio, Beijing, China) staining. Staining was imaged with Mshot inverted fluorescence microscope (Guangzhou, China).

### 4.9. Apoptosis Assay [39]

Annexin V/PI co-staining method was used to detect the degree of apoptosis. Cells were (1 × 10^6^/well) were coated at 6-well plates. After overnight and treating for 48 h, cells were digest with trypsin without EDTA. Then according to the kit (BD Biosciences, San Jose, CA, USA) instructions, 100 μL binding buffer was added and dyed in the dark. Finally flow cytometry (Amnis, Seattle, WA, USA) was used by analyzing.

### 4.10. Protein Preparation and TMT Labeling [40]

SW1990 cells were treated with 40 μM of Carabrone for 24 h. 300 μL RIPA Lysis and Extraction Buffer (Thermo Fisher Scientific, Waltham, MA, USA) were added to the sample and homogenized. Protein concentration was determined by Pierce™ BCA Protein Assay Kit (Thermo Fisher Scientific, Waltham, MA, USA). 25 μg peptides were diluted with 100 mM TEAB (Thermo Fisher Scientific, Waltham, MA, USA) for a final volume of 25 μL. To each sample tubes, 20 μL of TMT labeling reagent was added and reaction was incubated for 1 h at room temperature. Subsequently, 1 μL of 5% hydroxylamine was added and incubated for 15 min to quench the reaction. 300 μL of 5% ACN (Fisher Chemical, Waltham, MA, USA) and 0.1% TEA were loaded in column to remove unreacted TMT reagent, and the samples were evaporated to dryness using vacuum centrifugation.

### 4.11. Nano LC-MS/MS Analysis [40]

The tryptic peptides were dissolved in solvent A (0.1% formic acid in water)and directly loaded onto chromatographic column RPLC C18 (1.9 μm particles, 150 μm × 15 cm). The gradient ranged from 4 to 95% sol-vent B (an aqueous solution containing 0.1% formic acid and 80% acetonitrile) over 120 min, from 4% to 10% B for 5 min, from 10% to 22% B for 80 min, from 22% to 40% B for 25 min, from 40% to 95% B for 5 min and from 95% to 95% B for 5 min, all at a constant flow rate of 600 nL/min on an EASY-nLC 1200 UPLC system (ThermoFisher Scientific, Waltham, MA, USA).

The peptides were subjected to ESI nanospray source followed by tandem mass spectrometry (MS/MS) in Orbitrap Eclipse (Thermo) coupled online to the UPLC. The electrospray voltage applied was 2.2 kV. The *m*/*z* scan range was 350 to 1500 for a full scan, and intact peptides were detected in the Orbitrap (Thermo Fisher Scientific, Waltham, MA, USA) at a resolution of 60,000. Peptides were then selected for MS/MS using the NCE setting as 36, and the fragments were detected in the Orbitrap at a resolution of 17,500. The data-dependent procedure that we performed alternated between one MS scan followed by 20 MS/MS scans with a 54 ms dynamic exclusion. Automatic gain control (AGC) was set at 25 × 10^4^. The fixed first mass was set to 100 *m*/*z*.

### 4.12. Bioinformatics Analysis and Data Analysis [40]

The raw MS files were analyzed and searched against uniprot-Homo sapiens protein database based on the species of the samples using Proteome Discoverer 2.5. The parameters were set as follows: the protein modifications were carbamidomethylation (C) (fixed), oxidation (M) (variable); the enzyme specificity was set to trypsin; the maximum missed cleavages were set to 2; the precursor ion mass tolerance was set to 20 ppm, and MS/MS tolerance was 20 ppm. Only high confident identified peptides were chosen for downstream protein identification analysis. Omicsbean (http://www.omicsbean.cn, accessed on 8 February 2022) was used to analyze the obtained proteomic data. In all, GO enrichment analysis including biological process (BP), cellular components (CC) and molecular functions (MF). Kyoto Encyclopedia of Genes and Genomes (KEGG) pathway analysis and Protein-Protein Interaction (PPI) analysis were used by revealed the relationship between genes and genes, proteins and proteins, with default confidence of 0.400.

### 4.13. Statistical Analysis

Data are expressed as the means ± SDs based on at least 3 independent experiments. Proteomic data statistical differences were determined via one-way analysis of variance using SPSS 25.0. Other data statistical differences were performed with Student’s *t*-test or one-way ANOVA using GraphPad Prism 8, and values of *p* < 0.05 were considered significant.

## 5. Conclusions

**Figure 7:**
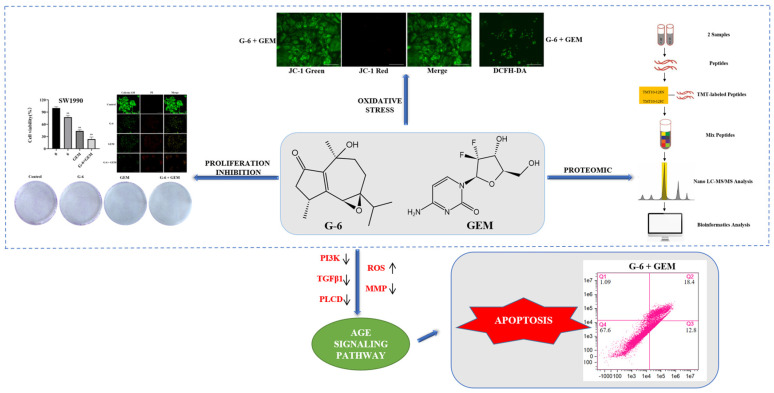
Schematic figure of the cancer inhibition mechanism of G-6 combined with GEM in SW1990 cells.

In summary, G-6, a naturally occurring guaiane-type sesquiterpenoid isolated from *Nardostachys jatamansi* DC., increased the susceptibility and inhibitory degree of GEM to the SW1990 pancreatic cancer cell lines, and the combined treatment of G-6 and GEM significantly inhibited the proliferation of SW1990 cells. Notably, G-6 combined with GEM regulated the AGE-RAGE signaling pathway by down-regulating PI3K, TGF*β*1 and PLCD, resulting in increased ROS, decreasing MMP, and finally inducing apoptosis in SW1990 cells (Figure 7).

## Data Availability

Data are contained within the article.

## Supplementary Materials

The following supporting information can be downloaded at: https://www.mdpi.com/article/10.3390/molecules27206849/s1, Figure S1: ^1^H NMR (400 MHz, CDCl_3_) spectrum of G-6; Figure S2: ^13^C NMR (100 MHz, CDCl_3_) spectrum of G-6; Figure S3: KEGG pathway enrichment map of AGE-RAGE signaling pathway; Table S1: List of up-regulated proteins in G-6 combined GEM-treated SW1990 cells; Table S2: List of down-regulated proteins in G-6 combined GEM-treated SW1990 cells.

## Sample Availability

Samples of the compounds are available from the authors.

## References

[1] Michl P., Gress T.M. (2013). Current concepts and novel targets in advanced pancreatic cancer. Gut.

[2] Vincent A., Herman J., Schulick R., Hruban R.H., Goggins M. (2011). Pancreatic cancer. Lancet.

[3] Neoptolemos J.P., Kleeff J., Michl P., Michl P., Costello E., Greenhalf W., Palmer D.H. (2018). Therapeutic developments in pancreatic cancer: Current and future perspectives. Nat. Rev. Gastroenterol. Hepatol..

[4] Kindler H.L., Niedzwiecki D., Hollis D., Sutherland S., Schrag D., Hurwitz H., Innocenti F., Mulcahy M.F., O’Reilly E., Wozniak T.F. (2010). Gemcitabine plus bevacizumab compared with gemcitabine plus placebo in patients with advanced pancreatic cancer: Phase III trial of the cancer and leukemia group B (CALGB 80303). J. Clin. Oncol..

[5] Gu Z., Du Y., Zhao X., Wang C.F. (2021). Tumor microenvironment and metabolic remodeling in gemcitabine—Based chemoresistance of pancreatic cancer. Cancer Lett..

[6] Li M. (2021). Clinical practice guidelines for the interventional treatment of advanced pancreatic cancer (5th edition). J. Inter. Med..

[7] Hani U., Osmani R.A.M., Siddiqua A., Wahab S., Batool S., Ather H., Sheraba N., Alqahtani A. (2021). A systematic study of novel drug delivery mechanisms and treatment strategies for pancreatic cancer. J. Drug Deliv. Sci. Technol..

[8] Pak P.J., Lee D.G., Sung J.H., Jung S.H., Han T.Y., Park S.H., Chung N. (2021). Synergistic effect of the herbal mixture C5E on gemcitabine treatment in PANC-1 cells. Mol. Med. Rep..

[9] Ke X., Wang Y., Xie Z., Liu Z.Q., Zhang C.F., Zhao Q. (2013). LY294002 enhances inhibitory effect of gemcitabine on proliferation of human pancreatic carcinoma PANC-1 cells. J. Huazhong Univ. Sci. Med..

[10] Yue Q., Gao G., Zou G., Yu H., Zheng X. (2017). Natural products as adjunctive treatment for pancreatic cancer: Recent trends and advancements. BioMed Res. Int..

[11] Chen Z., Zhang C., Gao F., Fu Q., Fu C., He Y. (2018). A systematic review on the rhizome of *Ligusticum chuanxiong* Hort. (Chuanxiong). Food Chem. Toxicol..

[12] Hashem S., Ali T.A., Akhtar S., Nisar S., Sageena G., Ali S., Al-Mannai S., Therachiyil L., Mir R., Elfaki I. (2022). Targeting cancer signaling pathways by natural products: Exploring promising anti-cancer agents. Biomed. Pharmacother..

[13] Liu Z., Wu X., Dai K., Li R., Zhang J., Sheng D., Ming-Yuen Lee S., Pak-Heng L.G., Zhou G.C., Li J.J. (2022). The new andrographolide derivative AGS-30 induces apoptosis in human colon cancer cells by activating a ROS-dependent JNK signalling pathway. Phytomedicine.

[14] Li W., Cao L., Chen X., Chen X., Lei J., Ma Q. (2016). Resveratrol inhibits hypoxia-driven ROS-induced invasive and migratory ability of pancreatic cancer cells via suppression of the Hedgehog signaling pathway. Oncol. Rep..

[15] Kaur H., Lekhak M.M., Chahal S., Goutam U., Jha P., Naidoo D., Ochattg S.J., Kumar V. (2020). *Nardostachys jatamansi* (D.Don) DC.: An invaluable and constantly dwindling resource of the Himalayas. S. Afr. J. Bot..

[16] Dhiman N., Bhattacharya A. (2020). *Nardostachys jatamansi* (D.Don) DC.-Challenges and opportunities of harnessing the untapped medicinal plant from the Himalayas. J. Ethnopharmacol..

[17] Ma L.M., Wang K., Meng X., Zheng Y.D., Wang C.B., Chai T., Naghavi M.R., Sang C.Y., Yang J.L. (2022). Terpenoids from *Nardostachys jatamansi* and their cytotoxic activity against human pancreatic cancer cell lines. Phytochemistry.

[18] Ahmad S., Khan H., Siddiqui Z., Khan M.Y., Rehman S., Shahab U., Godovikova T., Silnikov V., Moinuddine (2018). AGEs, RAGEs and s-RAGE; friend or foe for cancer. Semin. Cancer. Biol..

[19] Krisanits B.A., Woods P., Nogueira L.M., Woolfork D.D., Lloyd C.E., Baldwin A., Frye C.C., Peterson K.D., Cosh S.D., Guo Q.J. (2022). Non-enzymatic glycoxidation linked with nutrition enhances the tumorigenic capacity of prostate cancer epithelia through AGE mediated activation of RAGE in cancer associated fibroblasts. Transl. Oncol..

[20] Shahab U., Ahmad M.K., Mahdi A.A., Mahdi A.A., Waseem M., Arif B., Moinuddin, Ahmad S. (2018). The receptor for advanced glycation end products: A fuel to pancreatic cancer. Semin. Cancer Biol..

[21] Tong L., Chuang C., Wu S., Zuo L. (2015). Reactive oxygen species in redox cancer therapy. Cancer Lett..

[22] Zou P., Zhang J., Xia Y., Kanchana K., Guo G., Chen W., Huang Y., Wang Z., Yang S.L., Liang G. (2015). ROS generation mediates the anti-cancer effects of WZ35 via activating JNK and ER stress apoptotic pathways in gastric cancer. Oncotarget.

[23] Wang L., Hu T., Shen J., Zhang L., Li L., Chan R.L., Li M.X., Wu W.K., Cho C.H. (2016). Miltirone induced mitochondrial dysfunction and ROS-dependent apoptosis in colon cancer cells. Life Sci..

[24] Song N., Ma J.Y., Hu W., Guo Y.W., Hui L., Aamer M., Ma J. (2021). Lappaconitine hydrochloride inhibits proliferation and induces apoptosis in human colon cancer HCT-116 cells via mitochondrial and MAPK pathway. ACTA Histochem..

[25] Sharifi-Rad J., Ozleyen A., Tumer B.T., Adetunji C., Omari N., Balahbib A., Taheri Y., Bouyahya A., Martorell M., Martins N. (2019). Natural products and synthetic analogs as a source of antitumor drugs. Biomolecules.

[26] Zhang R., Tang C.P., Liu H.C., Ren Y.M., Ke C.Q., Yao S., Cai Y.Y., Zhang N.X., Ye Y. (2019). Tetramerized sesquiterpenoid ainsliatetramers A and B from ainsliaea f ragrans and their cytotoxic activities. Org. Lett..

[27] Lindenmeyer M.T., Hrenn A., Kern C., Castro V., Murillo R., Mu¨ller S., Laufer S., Schulte-Mönting J., Siedlea B., Merfort I. (2006). Sesquiterpene lactones as inhibitors of IL-8 expression in HeLa cells. Bioorg. Med. Chem..

[28] Su L.H., Zhang X.T., Ma Y.B., Geng C.G., Huang X.Y., Hu J., Li T.Z., Tang S., Shen C., Gao Z. (2021). New guaiane-type sesquiterpenoid dimers from *Artemisia atrovirens* and their antihepatoma activity. Acta Pharm. Sin. B.

[29] Zhao C., Gao W., Chen T. (2014). Synergistic induction of apoptosis in A549 cells by dihydroartemisinin and gemcitabine. Apoptosis.

[30] Yang S., Zhang D., Shen N., Wang G., Tang Z., Chen X. (2019). Dihydroartemisinin increases gemcitabine therapeutic efficacy in ovarian cancer by inducing reactive oxygen species. J. Cell Biochem..

[31] Pellegrini E., Multari G., Gallo F.R., Vecchiotti D., Zazzeroni F., Condello M., Meschini S. (2022). A natural product, voacamine, sensitizes paclitaxel-resistant human ovarian cancer cells. Toxicol. Appl. Pharmacol..

[32] Pomeroy A.E., Schmidt E.V., Sorger P.K., Palmer A.C. (2022). Drug independence and the curability of cancer by combination chemotherapy. Trends Cancer.

[33] Zhong X., Chen Z., Snovida S., Liu Y., Rogers J.C., Li L. (2015). Capillary electrophoresis-electrospray ionization-mass spectrometry for quantitative analysis of glycans labeled with multiplex carbonyl-reactive tandem mass tags. Anal. Chem..

[34] Wrzeszczynski K.O., Varadan V., Kamalakaran S., Levine D.A., Dimitrova N., Lucito R. (2013). Integrative prediction of gene function and platinum-free survival from genomic and epigenetic features in ovarian cancer. Methods Mol. Biol..

[35] Trachootham D., Alexandre J., Huang P. (2009). Targeting cancer cells by ROS-mediated mechanisms: A radical therapeutic approach?. Nat. Rev. Drug Discov..

[36] Liu C., Gong K., Mao X., Li W. (2011). Tetrandrine induces apoptosis by activating reactive oxygen species and repressing Akt activity in human hepatocellular carcinoma. Int. J. Cancer.

[37] Brennand K., Savas J.N., Kim Y., Tran N., Simone A., Hashimoto-Torii K., Beaumont K.G., Kim H.J., Topol A., Ladran I. (2015). Phenotypic differences in hiPSC NPCs derived from patients with schizophrenia. Mol. Psychiatry..

[38] Kumar P., Nagarajan A., Uchil P.D. (2018). Analysis of Cell Viability by the MTT Assay. Cold Spring Harb. Protoc..

[39] Chen H., Pan H., Qian Y., Zhou W.B., Liu X.A. (2018). MiR-25-3p promotes the proliferation of triple negative breast cancer by targeting BTG2. Mol. Cancer.

[40] Zheng Y.D., Zhang Y., Ma J.Y., Sang C.Y., Yang J.L. (2022). A Carabrane-Type Sesquiterpenolide Carabrone from Carpesium cernuum Inhibits SW1990 Pancreatic Cancer Cells by Inducing Ferroptosis. Molecules.

