# Supplementary material for: Nardoguaianone L Isolated from Nardostachys jatamansi Improved the Effect of Gemcitabine Chemotherapy via Regulating AGE Signaling Pathway in SW1990 Cells
Source: Molecules. 2022 Oct 13;27(20):6849. doi: 10.3390/molecules27206849 (PMC9610730; doi:10.3390/molecules27206849)
Supplement: Supplementary file 1 [file molecules-27-06849-s001.zip › Table S1 and Table S2.pdf]

Table S1. List of up-regulated proteins in **G-6** combined GEM-treated SW1990 cells

ID(Uniprot)	Gene name	Protein name	Fold change
A0A3B3IT88	VPS13C	Vacuolar protein sorting-associated protein 13C	5.685668206
Q8N3Y1	FBXW8	F-box/WD repeat-containing protein 8	5.683877781
D6RD66	WDR1	WD repeat-containing protein 1	4.536834596
A0A0A0MSI5	TANGO2	Transport and Golgi organization protein 2 homolog	4.245203364
Q7L3T8	PARS2	Probable proline--tRNA ligase, mitochondrial	3.872817104
A0A7I2V4C2	EIF3I	Eukaryotic translation initiation factor 3 subunit I	3.102592974
O14576	DYNC1I1	Cytoplasmic dynein 1 intermediate chain 1	2.953203005
A0A6I8PR93	DNAJC7	DnaJ homolog subfamily C member 7	2.881422306
R4GNB9	TRIM11	E3 ubiquitin-protein ligase TRIM11	2.853654708
H0YHL6	MARS	Methionine--tRNA ligase, cytoplasmic	2.704250127
P43250	GRK6	G protein-coupled receptor kinase 6	2.651390171
K7EJ78	RPS15	40S ribosomal protein S15	2.609541049
Q6Q0C0	TRAF7	E3 ubiquitin-protein ligase TRAF7	2.510799581
P35789	ZNF93	Zinc finger protein 93	2.384598068
A0A0C4DG98	THOC2	THO complex subunit 2	2.278039953
H7C561	SF1	Splicing factor 1	2.257606478
Q96DX7	TRIM44	Tripartite motif-containing protein 44	2.181868791
B7Z757	TFEC	Transcription factor EC	2.105777619
B7Z1U7	RBFOX1	RNA binding protein fox-1 homolog 1	2.081859574
H0Y4B9	PCCA	Propionyl-CoA carboxylase	2.059664038
K7EP67	EFTUD2	116 kDa U5 small nuclear ribonucleoprotein component	2.007842466

Table S2. List of down-regulated proteins in **G-6** combined GEM -treated SW1990 cells

ID(Uniprot)	Gene name	Protein name	Fold change
V9GYE8	SRR	Serine racemase	0.499573153
C9JI87	VDAC1	Voltage-dependent anion-selective channel protein 1	0.497083912
P23528	CFL1	Cofilin-1	0.496932572
P62851	RPS25	40S ribosomal protein S25	0.496923108
A0A2R8Y7Y3	EPB41	Protein 4.1	0.495287169
G5E9M4	ZNF277	Zinc finger protein 277	0.487839067
F6TDL0	LOC110117498-PIK3R3	P3R3URF-PIK3R3 readthrough	0.486428535
Q9UJN7	ZNF391	Zinc finger protein 391	0.486422116
A0A7I2YQL8	TGFB1	Transforming growth factor beta-1 proprotein	0.486415254
Q8N567	ZCCHC9	Zinc finger CCHC domain-containing protein 9	0.486292645
Q9NPF2	CHST11	Carbohydrate sulfotransferase 11	0.484874231
O14604	TMSB4Y	Thymosin beta-4, Y-chromosomal	0.479951613
Q86V71	ZNF429	Zinc finger protein 429	0.477910134
J3QL02	CNDP2	Cytosolic non-specific dipeptidase	0.470002076
A6NMY6	ANXA2P2	Putative annexin A2-like protein	0.459897031
P02689	PMP2	Myelin P2 protein	0.457716148
A0A1W2PP91	PIGT	GPI transamidase component PIG-T	0.455427098
P84243	H3F3B	Histone H3.3	0.452652347
Q9UNQ0	ABCG2	Broad substrate specificity ATP-binding cassette transporter ABCG2	0.45212888
H0Y7E6	EIF3L	Eukaryotic translation initiation factor 3 subunit L	0.452018786
E9PS65	HSPA8	Heat shock cognate 71 kDa protein	0.45162367
P52564	MAP2K6	Dual specificity mitogen-activated protein kinase kinase 6	0.449261454
Q99879	HIST1H2BM	Histone H2B type 1-M	0.4453084
P14209	CD99	CD99 antigen	0.443274247
Q6UXN9	WDR82	WD repeat-containing protein 82	0.436340538
Q9UQN3	CHMP2B	Charged multivesicular body protein 2b	0.432300601
A0A0U1RRG6	SMARCA2	Probable global transcription activator SNF2L2	0.424174601
H7C0P7	CCNYL1	Cyclin-Y-like protein 1	0.416512699
A0A087WZ65	ANK3	Ankyrin-3	0.416252229
H0YI26	USP15	Ubiquitin carboxyl-terminal hydrolase 15	0.414868315
B7Z509	GLS	Glutaminase	0.406460198
P51178	PLCD1	1-phosphatidylinositol 4,5-bisphosphate phosphodiesterase delta-1	0.405065054
A0A5F9ZHL5	ANK2	Ankyrin-2	0.403592711
B1AHM7	FBLN1	Fibulin-1	0.403418448
I3L4Y1	ABR	Active breakpoint cluster region-related protein	0.396544053
J3KNK1	MAPK9	Stress-activated protein kinase JNK	0.396016947
C9J296	LAMB1	Laminin subunit beta-1	0.395278142
A0A0C4DFP4	NGLY1	Peptide-N(4)-(N-acetyl-beta-glucosaminyl)asparagine amidase	0.387706361
E5RIA1	GPAT4	Glycerol-3-phosphate acyltransferase 4	0.385442289
H0YMR5	ZFAND6	AN1-type zinc finger protein 6	0.385091616

Q9NZ01	TECR	Very-long-chain enoyl-CoA reductase	0.384971138
P60484	PTEN	Phosphatidylinositol 3,4,5-trisphosphate 3-phosphatase and dual-specificity protein phosphatase PTEN	0.369367392
A0A0U1RQX0	TXNRD2	Thioredoxin reductase 2, mitochondrial	0.358565117
P16104	H2AFX	Histone H2AX	0.358408376
M0QXK2	SNRPA	U1 small nuclear ribonucleoprotein A	0.357947903
Q5QNY5	PEX19	Peroxisomal biogenesis factor 19	0.354766682
D6RCL2	MAP1B	Microtubule-associated protein 1B	0.350290378
H3BNL6	PDXDC1	Pyridoxal-dependent decarboxylase domain-containing protein 1	0.329118048
C9J191	KLHL22	Kelch-like protein 22	0.321817899
A0A3B3ISH4	PPP1R12A	Protein phosphatase 1 regulatory subunit 12A	0.315659244
C9JIF3	RETREG2	Reticulophagy regulator 2	0.281033076
E9PKF6	PPP6R3	Serine/threonine-protein phosphatase 6 regulatory subunit 3	0.278478557
H0Y7L7	PITRM1	Presequence protease, mitochondrial	0.243332408
J3KNC0	GTF2A1	Transcription initiation factor IIA subunit 1	0.229498171
C9J5J4	LIN9	Protein lin-9 homolog	0.21081521
P22694	PRKACB	cAMP-dependent protein kinase catalytic subunit beta	0.206100951
H0Y9G6	MRPL3	39S ribosomal protein L3, mitochondrial	0.186145873
A0A1B0GWJ6	MYO18A	Unconventional myosin-XVIIIa	0.171376551
H0Y6D7	TENT5A	Polynucleotide adenylyltransferase	0.125090992
A0A1B0GV73	ARHGAP21	Rho GTPase-activating protein 21	0.101474853
