# Supplementary figures and images for: Nardoguaianone L Isolated from Nardostachys jatamansi Improved the Effect of Gemcitabine Chemotherapy via Regulating AGE Signaling Pathway in SW1990 Cells
Source: Molecules. 2022 Oct 13;27(20):6849. doi: 10.3390/molecules27206849 (PMC9610730; doi:10.3390/molecules27206849)

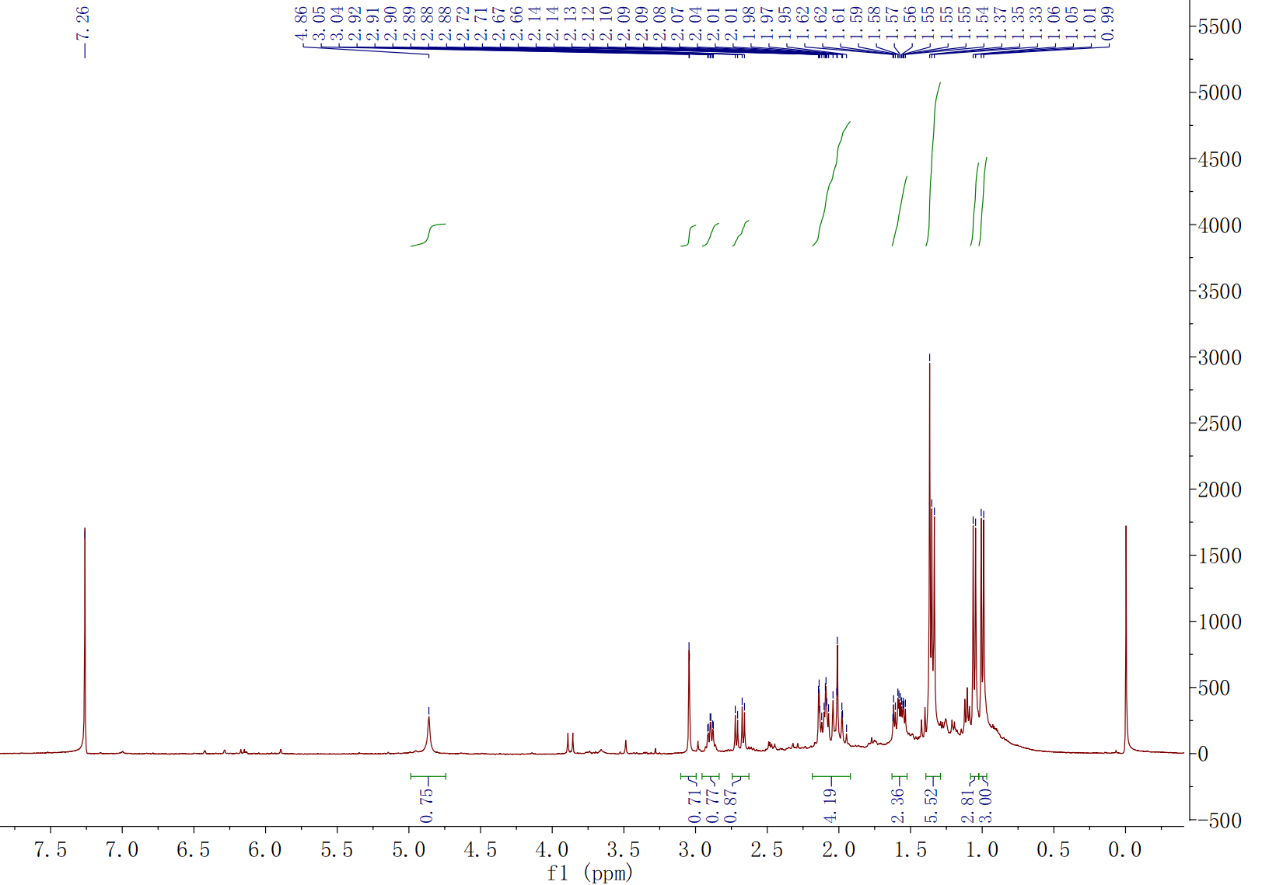

Supplement: Supplementary file 1 [file molecules-27-06849-s001.zip › Figure S1 1H NMR (400 MHz, CDCl3) spectrum of G-6.png]

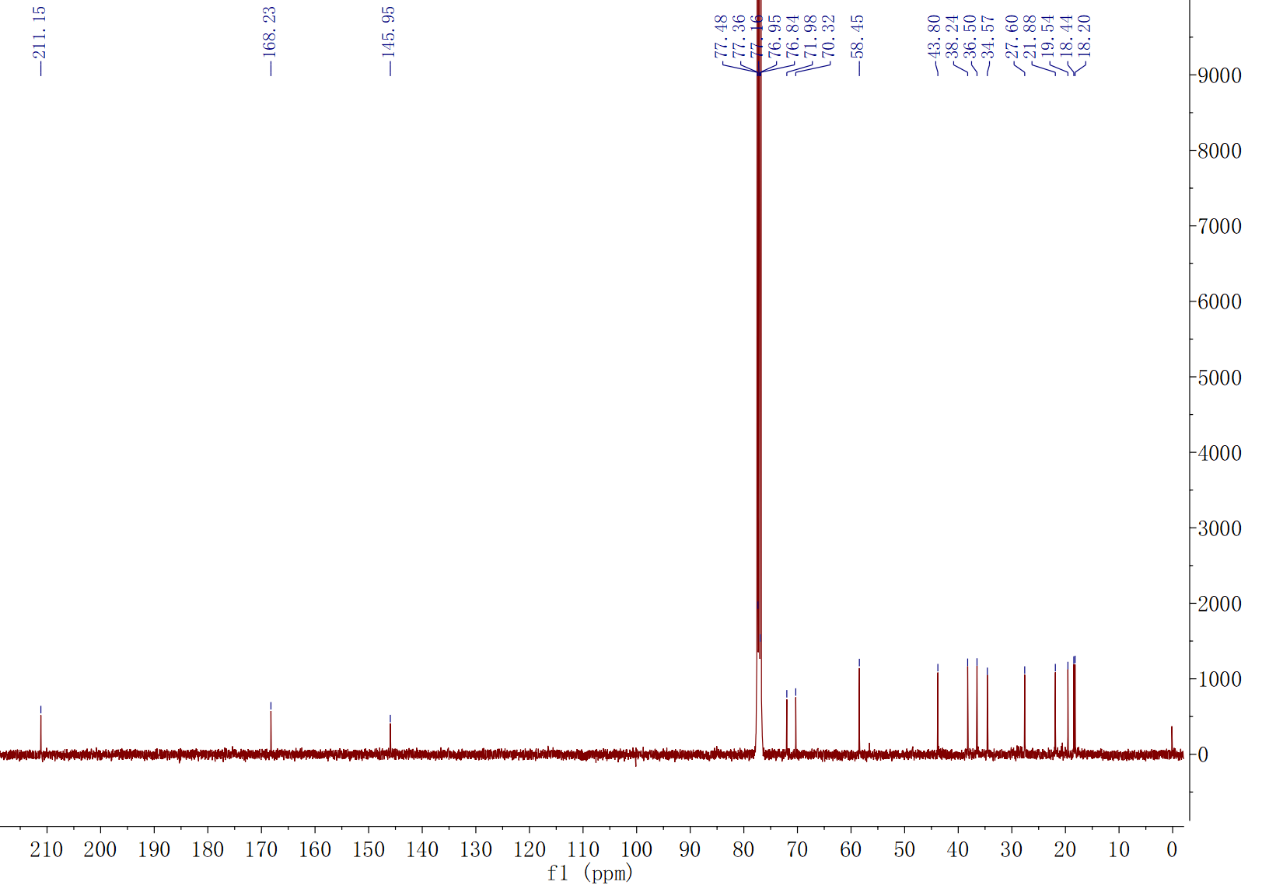

Supplement: Supplementary file 1 [file molecules-27-06849-s001.zip › Figure S2 13C NMR (100 MHz, CDCl3) spectrum of G-6.png]

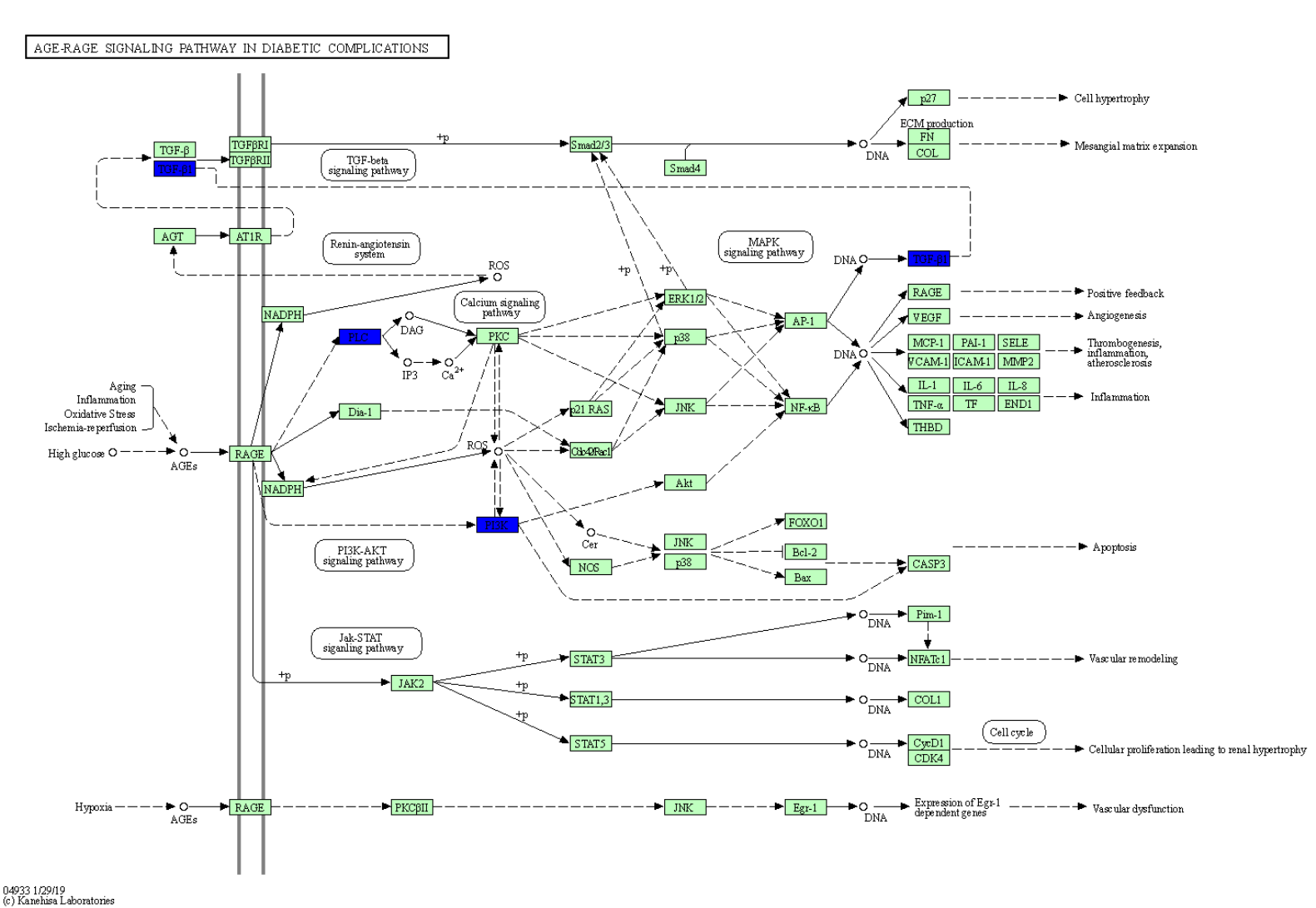

Supplement: Supplementary file 1 [file molecules-27-06849-s001.zip › Figure S3 KEGG pathway enrichment map of AGE-RAGE signaling pathway.png]
